# A Portable Pen-Shaped Otoscope for Telemedicine and Office-Based Otologic Examination: Feasibility and Patient Acceptability

**DOI:** 10.3390/jcm15031028

**Published:** 2026-01-27

**Authors:** Nao Hesaka, Takara Nakazawa, Seiji Kakehata

**Affiliations:** Endoscopic Ear Surgery Center, Ota General Hospital, Kawasaki 210-0024, Kanagawa, Japan; nao.jikeient@gmail.com (N.H.); takara.ogh@gmail.com (T.N.)

**Keywords:** pen-shaped otoscope, otoscopy, tympanic membrane, diagnostic imaging, patient-reported outcomes, telemedicine

## Abstract

**Background/Objectives**: Otoscopic examination is essential for the evaluation of ear diseases; however, conventional diagnostic devices have limitations related to portability, cost, and patient comfort. This study aimed to evaluate the feasibility and diagnostic performance of a newly developed pen-shaped otoscope compared with conventional otologic examination equipment. **Methods**: In this prospective study, 19 patients (28 ears) who underwent otologic examination at a tertiary referral center between April and June 2024 were included. Images of the external auditory canal and tympanic membrane were obtained using a pen-shaped otoscope, a video endoscope, and a microscope. Visualization of key tympanic membrane structures was assessed by physicians, and patients completed questionnaires evaluating pain, fear, image quality, and understanding of their disease. This prospective pilot feasibility study assessed the safety, usability, and preliminary diagnostic performance of the device. **Results**: Visualization rates of tympanic membrane structures using the pen-shaped otoscope, video endoscope, and microscope, respectively, were as follows: annulus tympanicus (57.1% vs. 89.3% vs. 9.1%), pars flaccida (89.3% vs. 96.4% vs. 45.5%), handle of the malleus (96.4% vs. 100% vs. 81.8%), and tympanic membrane vasculature (89.3% vs. 100% vs. 100%). No patients reported pain with the pen-shaped otoscope, whereas one patient reported pain with the video endoscope. Despite slightly lower image quality and disease understanding scores, several patients preferred the pen-shaped otoscope because of its ease of use and lack of discomfort. **Conclusions**: The pen-shaped otoscope provided clear visualization of key tympanic membrane structures, albeit with slightly lower image quality than the endoscope, while demonstrating high safety, portability, and ease of use. Its markedly lower cost supports its potential utility in smaller hospitals, outpatient clinics, and telemedicine applications. Further validation in larger cohorts and pediatric populations is warranted.

## 1. Introduction

In the post-COVID-19 era, concerns regarding infection risk associated with travel and in-person consultations have contributed to a decline in outpatient visits, while simultaneously accelerating the adoption of telemedicine and the development of related technologies. In otolaryngology, however, clinical practice remains highly dependent on physical examination using specialized equipment, which has limited the widespread implementation of telemedicine. Analyses of insurance reimbursement data have demonstrated that the annual growth of telemedicine utilization in otolaryngology lags behind that of other specialties, such as psychiatry and dermatology [1].

Nevertheless, a 2023 systematic review of telemedicine in otolaryngology concluded that video otoscopes are among the most validated tools for remote assessment in this field [2], indicating that otoscopy is particularly well suited for telemedicine applications. However, currently available video otoscopes have several limitations. Device insertion may pose a risk of trauma to the external auditory canal or tympanic membrane, and image quality or field of view may be suboptimal [3,4]. These shortcomings highlight the need for safer and higher-performance otoscopic devices.

In parallel with these technological challenges, Japan faces a growing structural imbalance in its healthcare workforce. Although the total number of physicians has increased nationwide, demand-adjusted physician supply has declined in many rural areas, reflecting a progressive shortage of physicians when population aging and healthcare needs are taken into account. Furthermore, longitudinal analyses have demonstrated that the geographic maldistribution of physicians has worsened over time, with an expanding disparity between urban and rural regions [5]. In this context, timely access to otolaryngology specialists for the diagnosis of middle ear diseases is increasingly limited in underserved communities, underscoring the need for reliable telemedicine-based diagnostic solutions.

We therefore hypothesized that a high-quality endoscopic device with a soft, deformable tip, designed for safe and easy handling, could enhance telemedicine by enabling otolaryngology specialists to be consulted remotely from community clinics or even patients’ homes. In this study, we report the first clinical evaluation of a novel pen-shaped otoscope and assess its feasibility and diagnostic performance in comparison with conventional otologic examination devices.

## 2. Materials and Methods

### 2.1. Ethical Approval

This study was approved by the Ethics Committee of Ota General Hospital (approval no. 23018) and conducted in accordance with the Declaration of Helsinki.

### 2.2. Patients

This prospective study included 19 patients examined at Ota General Hospital between April and June 2024. Informed consent was obtained from all participants. For patients younger than 20 years, written consent was provided by a parent or guardian, and assent was obtained from the patient after age-appropriate explanation.

### 2.3. Devices

**Figure 1:**
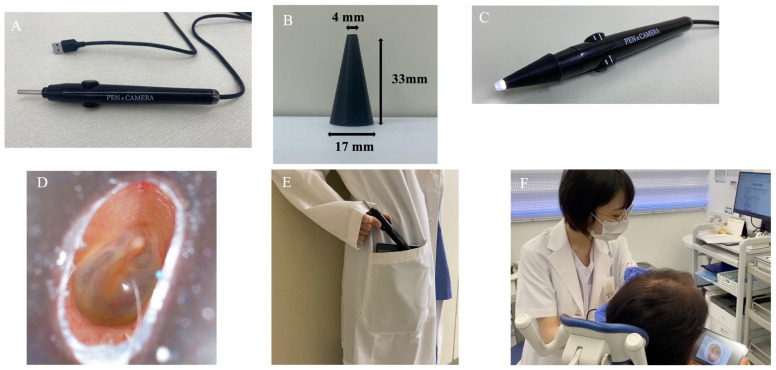
Features of the pen-shaped otoscope. (**A**) Main unit and USB cable. (**B**) Dimensions of the silicone tip. (**C**) The silicone tip attached to the distal end for ear examination. (**D**) The silicone tip deforms to conform to the shape of the external auditory canal. (**E**) The pen-shaped otoscope and tablet are compact enough to fit into a lab coat pocket. (**F**) Patients viewing real-time video images on a handheld device.

Ear examinations were performed using three devices: The pen-shaped otoscope (PEN CAMERA^®^ PE1, Koden Corporation, Tokyo, Japan) has a total length of 18 cm, a body width of 2.3 cm, and a weight of 82 g (Figure 1A). The combined camera and light source unit measures 3 cm in length and 4 mm in diameter, incorporating a 1-million–pixel imaging sensor with a depth of focus of 5–50 mm and a 100° field of view. A newly developed conical silicone tip, which is soft and deforms to conform to the shape of the external auditory canal, was designed for improved usability and attached to the distal end of the device (Figure 1B–D). Images and videos were captured via cable connection to a tablet. Both the otoscope and tablet were small enough to fit into a laboratory coat pocket (Figure 1E). Patients could view real-time videos of their own ears on handheld devices (Figure 1F). Still images were recorded using a TECLASTPAD P85T tablet (Guangzhou Shangke Information Technology Co., Ltd., Guangzhou, China). The main unit is commercially available in Japan; however, the conical silicone tip was newly developed for this study. The device was used for clinical evaluation under institutional ethics approval and written informed consent.

Images were also obtained using a video endoscope (VISERA ELITE II^®^, ENF-VH^®^, Olympus Corporation, Tokyo, Japan), which has a distal outer diameter of 3.2 mm and incorporates a 1-million–pixel sensor with a 110° field of view and a depth of focus of 5–50 mm. Microscopic images were captured using an operating microscope (OPMI PICO^®^, Zeiss Corporation, Oberkochen, Germany) equipped with an integrated HD camera (2 million pixels) providing HDMI output. During microscopic examination, either a standard otoscope speculum or a transparent plastic speculum was used to obtain an adequate view of the ear canal and tympanic membrane. All images were subsequently stored in the electronic medical record system (CITA, FUJIFILM Medical Co., Ltd., Tokyo, Japan).

### 2.4. Examination Procedure

All examinations were performed in the outpatient clinic, where all three devices were used on the same day for each patient by the same examiner. Multiple still images of the external auditory canal and tympanic membrane were obtained with each device. Microscope images were evaluated only in cases in which transparent specula were used, allowing visualization of the tympanic membrane through the speculum. As a result, microscope-based evaluation was available in a subset of ears (*n* = 11), whereas data from the pen-shaped otoscope and endoscope were obtained in all examined ears (*n* = 28).

### 2.5. Physician Evaluation

**Figure 2:**
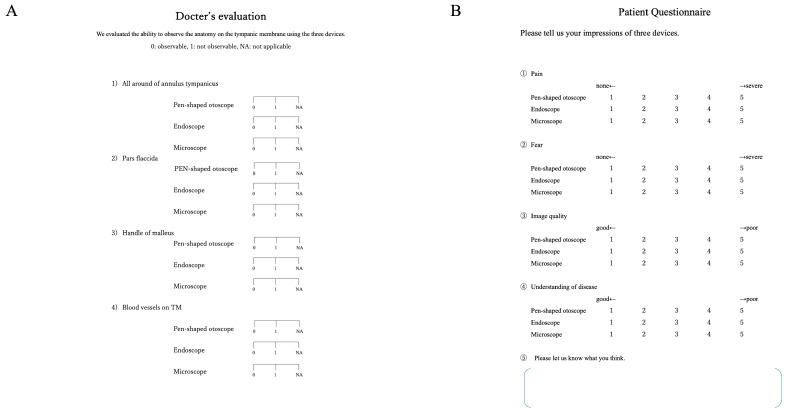
Physician evaluation and patient questionnaire. (**A**) Physicians evaluated the ability of the three devices to visualize tympanic membrane anatomy using a standardized assessment sheet. Scores were defined as follows: 0, observable; 1, not observable; NA, not applicable. (**B**) Patients rated each device using a 5-point scale for pain, fear, image quality, and understanding of their disease. Pain and fear were scored from 1 (none) to 5 (severe), whereas image quality and understanding were scored from 1 (good) to 5 (poor). For all questionnaire items, higher scores indicate worse outcomes (i.e., more pain/fear and poorer image quality/understanding).

To allow for a standardized comparison, an evaluation sheet was prepared (Figure 2A). Two otolaryngologists, blinded to the examiner, independently reviewed a series of still images for each case, rather than a single image. Based on the overall assessment of these images, they evaluated the visibility of key structures, including the annulus tympanicus, pars flaccida, handle of the malleus, and tympanic membrane vasculature. Scores were defined as follows: 0, observable; 1, not observable; NA, not applicable. In patients with tympanostomy tubes, the ability to visualize the circumference and lumen of the tube was evaluated.

Inter-rater reliability between the two evaluators was assessed using Cohen’s kappa (κ) statistic. In cases of disagreement, the final judgment was determined by consensus following joint review and discussion of the image set. The annulus tympanicus was judged as observable when the entire circumference of the tympanic annulus could be clearly identified. The pars flaccida was judged as observable when the entire flaccid portion could be fully visualized. The handle of the malleus was judged as observable when the malleus handle, or at least the adjacent tympanic membrane in its expected location, was completely visualized. The tympanic membrane vasculature was judged as observable when any portion of the vascular structures on the tympanic membrane could be clearly identified. In patients with an open mastoid cavity, visibility of the entire cavity was assessed.

### 2.6. Patient Evaluation

Patients completed a questionnaire (Figure 2B) to assess their experience with each device (pen-shaped otoscope, endoscope, and microscope). They rated pain and fear on a 5-point scale ranging from 1 (none) to 5 (severe), and image quality and understanding of their condition from 1 (good) to 5 (poor). A free-text section was also included to allow for additional comments.

### 2.7. Statistical Analysis

Three otologic examination devices (endoscope, pen-shaped otoscope, and microscope) were compared. Physician-assessed visualization of tympanic membrane structures was analyzed using Fisher’s exact test, while patient-reported outcomes were analyzed using the Friedman test. When the overall analysis indicated a significant difference among the three devices, post hoc pairwise comparisons were performed to identify differences between device pairs, with appropriate adjustment for multiple comparisons. For post hoc analyses, adjustment for multiple comparisons was performed using the Holm method. Accordingly, results are interpreted with caution, and conclusions are limited to descriptive or exploratory comparisons rather than formal equivalence between devices. All statistical analyses were performed using R (version 4.5.2). Because this study was intended as a first-in-human pilot feasibility study, formal sample size calculation was not performed. The sample size was determined pragmatically to evaluate safety, usability, and preliminary diagnostic performance rather than to establish diagnostic equivalence between devices.

## 3. Results

### 3.1. Patient Characteristics

Nineteen patients (28 ears) were included in the study. The median age was 52 years (range, 8–79 years), with 6 males and 13 females. Twenty-four ears had middle ear disease, including chronic otitis media, adhesive otitis media, and cholesteatoma. Seven ears had normal tympanic membranes, including cases of otosclerosis and congenital malformation. Detailed disease characteristics are summarized in Table 1.

### 3.2. Physician Evaluation

All 28 ears were examined using the pen-shaped otoscope and the endoscope. Microscope images were available for evaluation only when obtained through transparent ear specula (*n* = 11); images obtained using metallic specula were excluded due to insufficient image quality. Inter-rater agreement assessed using Cohen’s kappa showed moderate agreement (κ = 0.45, *p* < 0.001), with a percent agreement of 75.7%.

### 3.3. Representative Images of the Ear Canal and Tympanic Membrane

**Figure 3:**
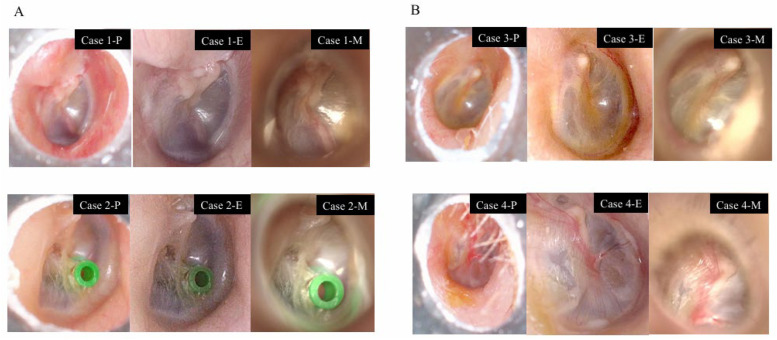
Comparison of the pen-shaped otoscope (P), endoscope (E), and microscope (M). All images are from the right ear. (**A**) Cases with good visualization: Case 1. Postoperative tympanic membrane findings following surgery for pars flaccida cholesteatoma. Case 2. Tympanic membrane after placement of a tympanic ventilation tube. (**B**) Cases where the pen-shaped otoscope was at a disadvantage: Case 3. Overhang of the anterior and inferior walls of the external auditory canal. Case 4. Presence of hair in the external auditory canal.

Representative images demonstrating similar visualization of the pen-shaped otoscope and the endoscope, as well as cases in which the pen-shaped otoscope was inferior, are shown in Figure 3A and Figure 3B, respectively.

Case 1 shows a postoperative ear following surgery for pars flaccida cholesteatoma, in which the scutum was reconstructed with cartilage. Case 2 demonstrates the tympanic membrane after placement of a tympanic ventilation tube. In Case 1, all key tympanic membrane structures were clearly visualized using all devices, whereas in Case 2, all key structures were clearly visualized with the pen-shaped otoscope and the endoscope, but not with the microscope.

Case 3 illustrates a situation in which both the pen-shaped otoscope and the microscope were at a disadvantage due to anterior wall overhang of the external auditory canal. In Case 4, the presence of hair within the external auditory canal prevented adequate visualization of the tympanic membrane using the pen-shaped otoscope.

### 3.4. Annulus Tympanicus

**Figure 4:**
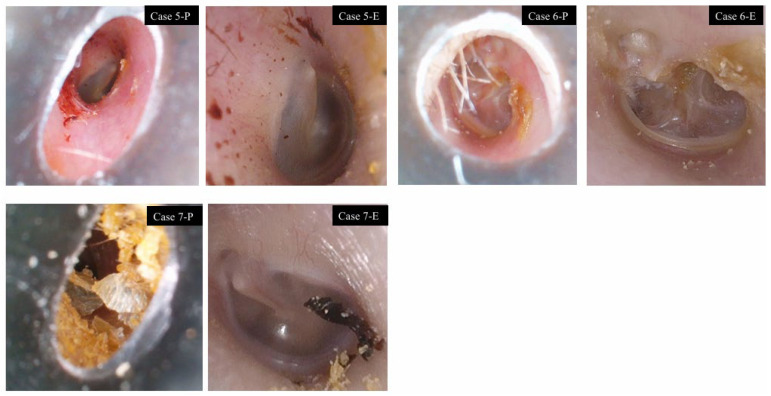
Cases with limited visualization of tympanic membrane vasculature using the pen-shaped otoscope. Case 5: Narrow external auditory canal (right ear). Case 6: Narrow external auditory canal with hair (left ear). Case 7: Narrow external auditory canal with cerumen (left ear).

**Figure 5:**
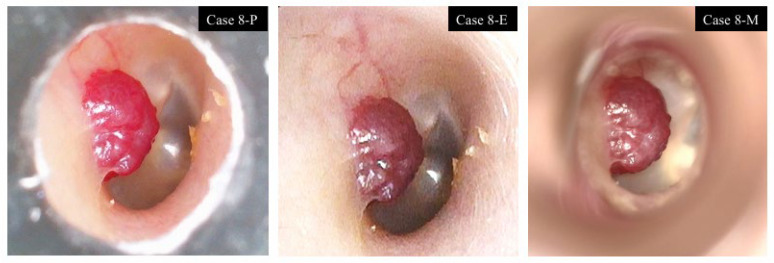
External auditory canal tumor. Case 8: Right ear. A well-circumscribed, reddish mass arising from the posterior wall of the external auditory canal. The tumor surface and feeding vessels were clearly visualized with all three devices.

Complete circumferential visualization of the annulus tympanicus was achieved in 57.1% of cases using the pen-shaped otoscope, 89.3% using the endoscope, and 9.1% using the microscope. All cases visualized with the endoscope but not with the pen-shaped otoscope or the microscope were attributable to overhang of the anterior or inferior external auditory canal wall. Even when multiple still images were reviewed, complete circumferential visualization with the microscope remained difficult because of its straight-line viewing geometry. In contrast, the pen-shaped otoscope offers a wide field of view, facilitating circumferential assessment. Complete visualization was not achieved with any device in some cases, due to severe canal wall overhang or the presence of an external auditory canal tumor (Figure 4 and Figure 5).

In the three-group comparison, a significant difference was observed in complete visualization of the annulus tympanicus (*p* = 0.000039). Visualization with the endoscope was significantly more frequent than with the pen-shaped otoscope (*p* = 0.021), and both the endoscope and the pen-shaped otoscope achieved significantly higher visualization rates than the microscope.

### 3.5. Pars Flaccida

Visualization rates were 89.3% with the pen-shaped otoscope, 96.4% with the endoscope, and 45.5% with the microscope. There was a case in which only the endoscope provided adequate visualization, owing to anterior canal wall overhang with adherent cerumen that obscured the view using the pen-shaped otoscope and the microscope. A significant difference in visualization of the pars flaccida was observed among the three devices (*p* = 0.00225). There was no significant difference between the endoscope and the pen-shaped otoscope, whereas both devices demonstrated significantly higher visualization rates than the microscope (*p* = 0.0026 and *p* = 0.0155, respectively).

### 3.6. Handle of Malleus

Visualization rates were 96.4% with the pen-shaped otoscope, 100% with the endoscope, and 81.8% with the microscope. In one ear with anterior canal wall overhang and adherent cerumen, both the pen-shaped otoscope and the microscope failed to achieve adequate visualization. No significant difference was detected among the three devices.

### 3.7. Tympanic Membrane Vasculature

Visualization rates were 89.3% with the pen-shaped otoscope, 100% with the endoscope, and 100% with the microscope. In all three cases in which the pen-shaped otoscope failed to visualize the vasculature, the cause was either a narrow external auditory canal alone, a narrow canal with hair, or a narrow canal with cerumen, which prevented the otoscope from reaching the optimal focal position (Figure 4).

No significant differences were observed among the three devices (Table 2). Overall, the pen-shaped otoscope showed similar trends in visualization to the endoscope across most evaluated structures, although visualization of the annulus tympanicus was lower.

### 3.8. Special Cases

In one case of an external auditory canal tumor, a well-circumscribed, reddish, pedunculated mass arising from the external auditory canal wall and partially obstructing the canal lumen was observed. The tumor surface and feeding vessels were clearly visualized with all three devices (Figure 5). In one postoperative ear with a canal wall–down mastoid cavity, complete visualization was difficult using the pen-shaped otoscope.

### 3.9. Patient Questionnaire

**Figure 6:**
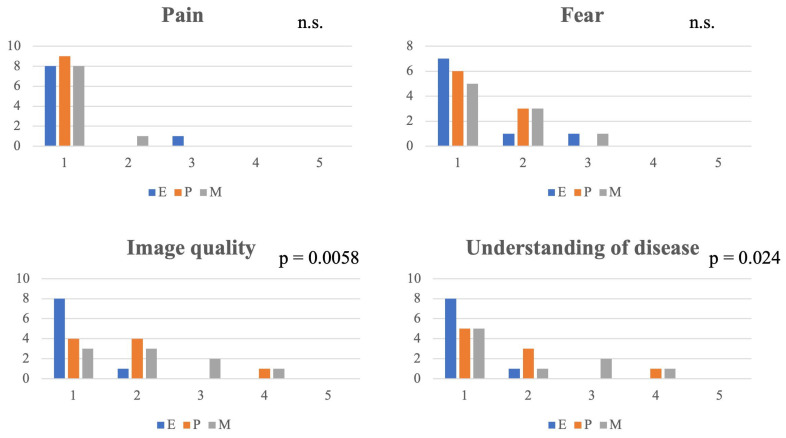
Comparison of patient-reported outcomes among the pen-shaped otoscope, endoscope, and microscope. Pain, fear, image quality, and understanding of disease were rated on a 5-point scale by nine patients for the pen-shaped otoscope (P), endoscope (E), and microscope (M). Group comparisons were performed using the Friedman test. Significant differences were observed for image quality (*p* = 0.0058) and understanding of disease (*p* = 0.024), whereas no significant differences were found for pain or fear. Post hoc pairwise comparisons did not reveal significant differences after adjustment for multiple testing. For all questionnaire items, higher scores indicate worse outcomes (i.e., more pain/fear and poorer image quality/understanding of disease).

Questionnaires were completed by 9 of 19 patients. Non-completion was primarily due to time constraints in routine clinical practice. Each patient rated pain, fear, image quality, and understanding of disease on a 5-point scale for all three devices. Results are summarized in Figure 6.

**Pain and Fear:** One patient reported pain with the endoscope and one with the microscope, whereas no patients reported pain with the pen-shaped otoscope. The Friedman test showed no significant differences among the devices for pain (*p* = 0.368) or fear (*p* = 0.607).
**Image Quality:** A significant difference in image quality was observed among the devices (*p* = 0.0058). The endoscope tended to receive higher ratings than both the pen-shaped otoscope and the microscope; however, no statistically significant differences were identified in post hoc pairwise comparisons after adjustment for multiple testing.
**Understanding of Disease:** A significant difference among the devices was also observed for understanding of disease (*p* = 0.024). However, post hoc analyses did not reveal any significant differences between individual devices.
**Patient comments:** Free-text responses included remarks such as, “I didn’t have to worry about going too far with the pen-shaped otoscope, and I think I could use it at home,” and “The images from the pen-shaped otoscope were easy to see, there was no discomfort, and I was able to concentrate on the explanation.”

## 4. Discussion

The COVID-19 pandemic accelerated the development of remote diagnostic tools, and a 2022 systematic review identified video otoscopes as the most suitable devices for telemedicine in otorhinolaryngology [1,2,3,4,5,6,7]. However, existing video otoscopes continue to present limitations related to safety, image quality, and field of view. Reported issues include suboptimal resolution and the potential for trauma to the external auditory canal or tympanic membrane due to narrow insertion tips. With the increasing demand for telemedicine, the development of safer and higher-performance devices for remote otologic evaluation is warranted.

### 4.1. Comparison with Conventional Devices and Diagnostic Performance

In the present study, the flexible video endoscope provided superior visualization owing to its thin diameter and wide field of view, allowing for clear observation despite the presence of hair, cerumen, or curvature of the external auditory canal. In addition, its flexibility allows for advancement along curved ear canals, enabling close positioning near the tympanic membrane. However, its high cost and limited portability restrict its routine use to specialist facilities.

The microscope demonstrated excellent visualization of fine structures in evaluable cases, including tympanic membrane vasculature, even in abnormal membranes. However, because the microscope relies on a straight-line view, visualization was constrained by canal anatomy and alignment. Despite its high resolution, the microscope’s narrow field of view, operator dependence, and poor portability limited its suitability for detailed image documentation in this study.

By comparison, the pen-shaped otoscope achieved visualization rates of approximately 90% for major tympanic membrane structures, except for the annulus tympanicus. Although slightly inferior to the endoscope in some respects, it enabled clear visualization of fine structures, including tympanic vessels. Visualization was limited in cases with canal wall overhang, hair, or cerumen, reflecting the design of the short conical silicone tip.

In addition, the pen-shaped otoscope is equipped with a light source with a width of approximately 4 mm, which limits close advancement toward the tympanic membrane. Previous anatomical studies have shown that the minimum diameter of the osseous external auditory canal ranges from approximately 3.4 to 6.6 mm, with mean values around 5 mm in both pediatric and adult populations [6]. When the canal diameter approaches the lower end of this range, the width of the light source alone occupies a substantial portion of the canal lumen, making it difficult to advance the otoscope tip sufficiently close to the tympanic membrane. This anatomical constraint likely contributes to reduced visualization in narrow or curved canals, particularly near the annulus tympanicus.

While visualization of the annulus tympanicus was limited compared with that achieved using a flexible endoscope, the level of visualization obtained with the pen-shaped otoscope appears sufficient for routine screening purposes, such as the evaluation of otitis media. Accordingly, the device should be positioned as a safe screening and triage tool for non-otolaryngologists rather than as a replacement for specialist diagnostic equipment. Future refinements, such as longer or thinner silicone tips and improved lens design, may help overcome these limitations.

Although the imaging sensor resolution is 1 megapixel, which is lower than that of surgical microscopes, it appears adequate for telemedicine-based screening and triage by generalists, where the primary objective is identification of gross abnormalities and determination of the need for specialist referral rather than definitive diagnosis.

### 4.2. Patient Perspective and Clinical Implications

**Figure 7:**
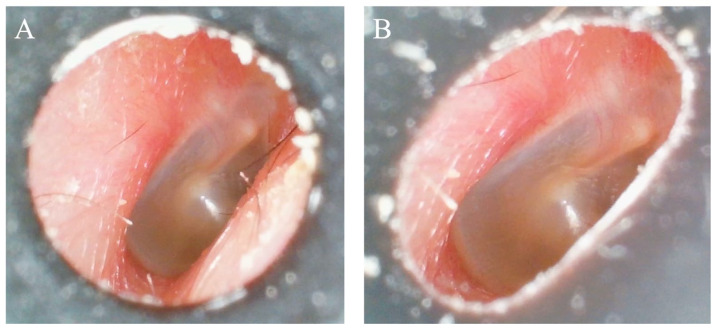
Features of the pen-shaped otoscope. Right ear. (**A**) A conventional rigid ear tip, which does not deform to conform to the shape of the external auditory canal. (**B**) The silicone tip of the pen-shaped otoscope, which deforms to fit the external auditory canal, allowing improved adaptability to individual anatomical variations.

Patient-reported outcomes indicated that the pen-shaped otoscope caused no pain, in contrast to one report of pain associated with the endoscope, underscoring the safety advantage of its silicone tip. The pen-shaped otoscope, equipped with a soft, deformable silicone tip and a relatively large-diameter integrated camera–light source unit (3 cm in length and 4 mm in diameter), acts as a physical safeguard against deep insertion, thereby enhancing device safety and reducing the risk of trauma to the external auditory canal (Figure 7).

Although image quality and disease understanding were rated slightly lower than with the endoscope, patients expressed a clear preference for the pen-shaped otoscope over the microscope and valued its comfort and ease of use. Several patients remarked that they could envision using the device at home. Given its portability, safety, and usability, the pen-shaped otoscope may facilitate remote consultations in clinics without otolaryngology specialists and enable home-based monitoring with remote expert interpretation. Previous studies have demonstrated that trained parents and non-specialists using smartphone-based otoscopes can achieve clinically meaningful diagnostic accuracy, including appropriate referral decisions [8,9,10,11,12]. Similar applications may be feasible with the pen-shaped otoscope.

Compared with smartphone-based otoscopes, the pen-shaped otoscope offers a dedicated integrated camera–light source and a soft, deformable silicone tip that may improve handling and safety. Smartphone-based systems are widely accessible and inexpensive, but their image quality and illumination may vary depending on the phone model and attachment design. In telemedicine, such devices may serve two complementary roles: initial screening and triage in primary-care or home settings, and remote specialist-supported diagnostic confirmation when high-quality tympanic membrane visualization is achievable.

Although the present study primarily included adult patients, two pediatric patients aged 8 years or older (three ears) were included. While younger children were not evaluated in this initial feasibility study, the soft, deformable silicone tip represents a particular advantage for pediatric ear examinations, in which patient movement and discomfort often limit safe visualization. In future primary care settings, this design may facilitate safer otoscopic screening in children by non-specialists, potentially improving early detection of otitis media and other common pediatric ear conditions.

### 4.3. Cost-Effectiveness and Future Perspectives

Compared with standard handheld otoscopes commonly used in primary care, the pen-shaped otoscope offers several advantages, including digital image acquisition, improved illumination, enhanced safety through its soft silicone tip, and the ability to share images for remote consultation. These features may provide a meaningful upgrade over conventional otoscopes while remaining far more affordable and portable than high-end endoscopic systems.

The endoscope system used in this study costs approximately 7.75 million yen (≈SD 52,700), whereas the pen-shaped otoscope and tablet together cost approximately 220,000 yen (≈USD 1500). This substantial cost difference, combined with adequate diagnostic performance, supports the potential adoption of the pen-shaped otoscope in smaller hospitals and outpatient clinics with limited access to high-cost equipment (Table 3).

Looking forward, integration with artificial intelligence may further enhance the telemedicine utility of the device. Previous studies have demonstrated the potential of AI-based analysis of tympanic membrane images, including GAN-generated synthetic data, smartphone-based diagnostic applications, and multimodal large-language-model approaches [13,14,15,16,17,18]. Application of similar AI technologies to images acquired with the pen-shaped otoscope may strengthen its role as a safe, portable, and accurate tool for telemedicine.

### 4.4. Limitations

This study has several limitations, including a small sample size. In addition, further validation in younger pediatric populations is required, as all participants in this study were aged ≥8 years. Given the pilot nature of this study and the limited sample size, the present findings should be interpreted as preliminary and require confirmation in larger, adequately powered studies. Another limitation of this study is that microscope-based evaluation was possible only in a subset of cases due to the use of transparent specula, resulting in unequal sample sizes across devices. Accordingly, comparisons involving the microscope should be interpreted with caution. In addition, patient-reported outcomes were available for only 47% of participants, primarily due to time constraints in routine clinical practice. Therefore, these results should be interpreted as preliminary and may be subject to selection bias. In addition, the order of device usage was not randomized, which may have introduced order-related bias; however, this reflects the pragmatic design of this preliminary feasibility study.

## 5. Conclusions

The newly developed pen-shaped otoscope demonstrated adequate diagnostic performance, achieving visualization rates of approximately 90% for major tympanic membrane structures. Although slightly inferior to the flexible endoscope in certain aspects, it provided clear images, caused no patient discomfort, and was strongly preferred over the microscope. Its portability, safety, ease of use, and markedly lower cost underscore its potential for adoption in smaller hospitals, outpatient clinics, and telemedicine settings. Further studies involving larger cohorts and younger pediatric populations, along with continued device refinements, are warranted to establish its diagnostic accuracy and clinical utility.

## Figures and Tables

**Table 1:** Otologic diagnoses of the examined ears.

Diagnosis	Number of Ears
Abnormal tympanic membrane	21
Adhesive otitis media (including atelectasis)	7
Chronic otitis media	7
Pars flaccida cholesteatoma	4
Otitis media with effusion	3
Eosinophilic otitis media	2
After surgery of canal wall down	1
Case of placement of a tympanic ventilation tube	3
Normal tympanic membrane	7
No disease	3
Otosclerosis	2
Congenital ossicular malformation	1
Sudden deafness	1
Tumor of the external auditory canal	1

**Table 2:** Physician-assessed visualization rates by device.

	Pen-Shaped Otoscope	Endoscope	Microscope	*p* Value
Annulus tympanicus ^†^	57.1% (16/28)	89.3% (25/28)	9.1% (1/11)	<0.001
Pars flaccida ^‡^	89.3% (25/28)	96.4% (27/28)	45.5% (5/11)	0.002
Handle of malleus	96.4% (27/28)	100.0% (28/28)	81.8% (9/11)	0.068
Tympanic membrane vasculature	89.3% (25/28)	100.0% (28/28)	100.0% (11/11)	0.205

^†^ Visualization was significantly better with the flexible endoscope than with the pen-shaped otoscope, and significantly better with the pen-shaped otoscope than with the microscope. ^‡^ Both the pen-shaped otoscope and the flexible endoscope showed significantly higher visualization rates than the microscope, with no significant difference between the two devices.

**Table 3:** Comparing device characteristics.

	Distal Diameter	Portability	Cost (Relative)	Safety/Suitability
Pen-shaped otoscope	Moderate (camera 4 mm)	High	Moderate	Soft silicone tip, limited deep insertion, suitable for routine examination and screening, video recording available
Video endoscope	Small (3.2 mm)	Moderate	High	Allows close approach to tympanic membrane, requires expertise
Microscope	Indirect (via speculum)	Low	Very high	Stable, hands-free visualization, specialist use
Conventional otoscope	Indirect (via speculum)	High	Low–moderate	Line-of-sight view, standard routine examination
Smartphone-based otoscopes	Small (~3–4 mm camera)	High	Low	Image capture and sharing via smartphone, consumer/clinical use varies

## Data Availability

The data presented in this study are available from the corresponding author upon reasonable request. The data are not publicly available due to ethical and privacy restrictions.
